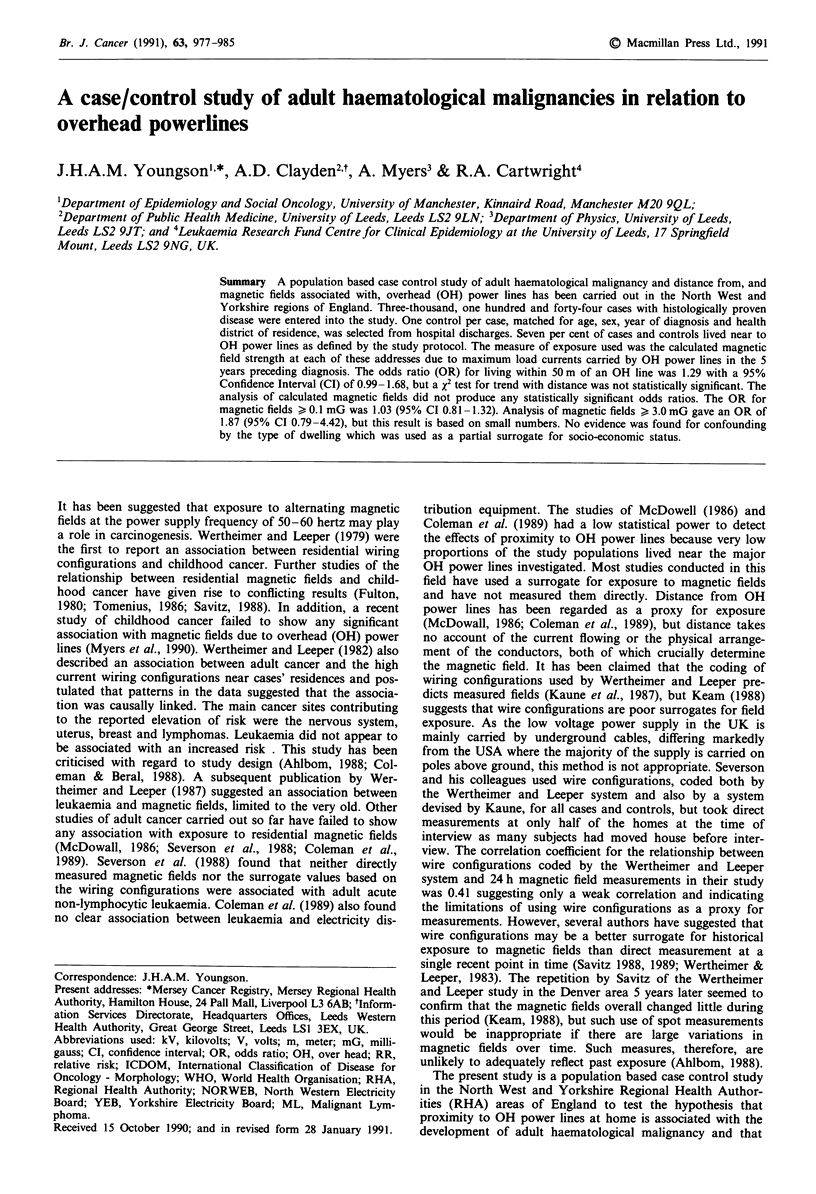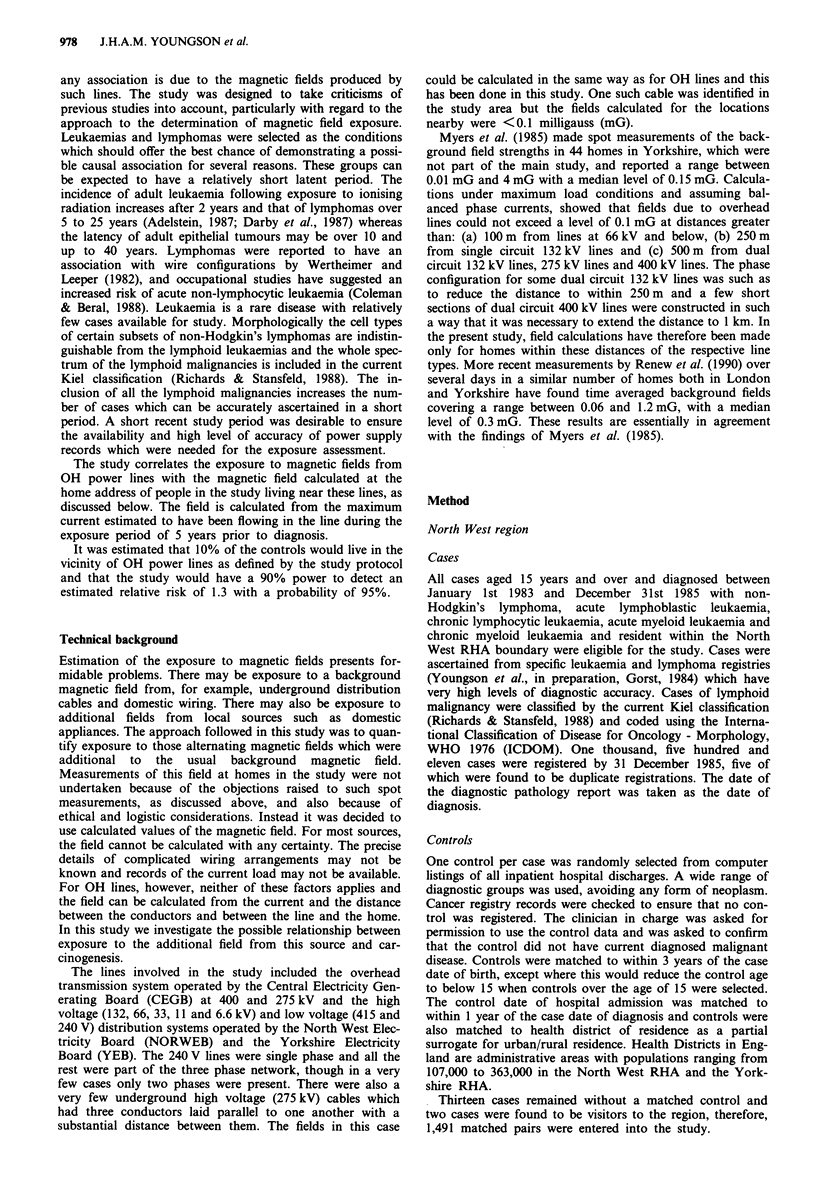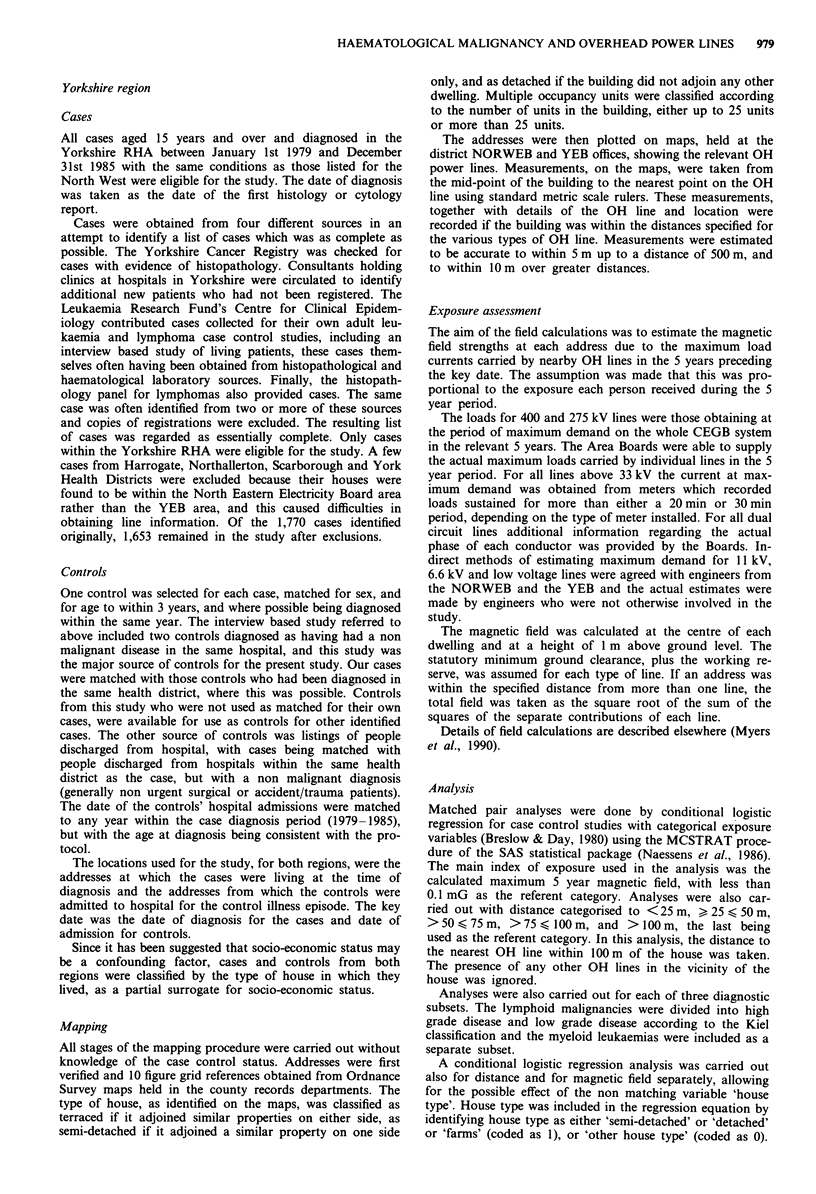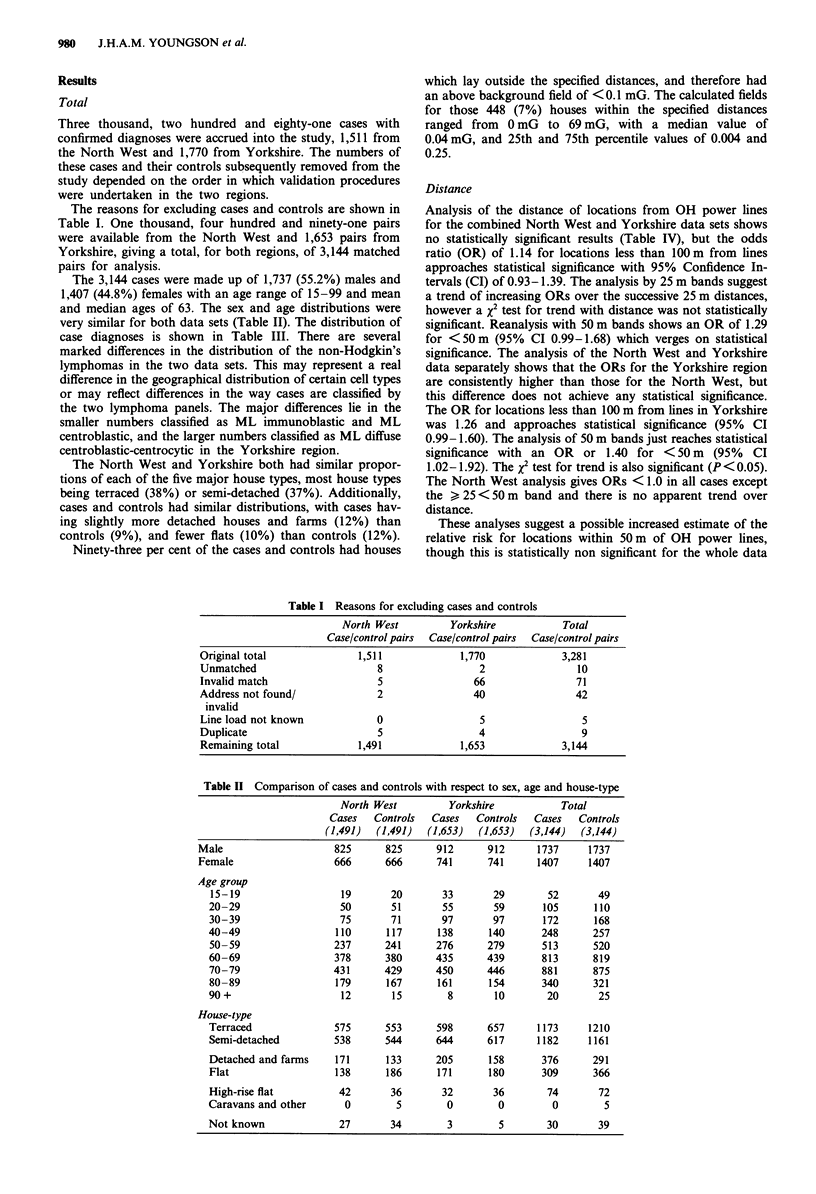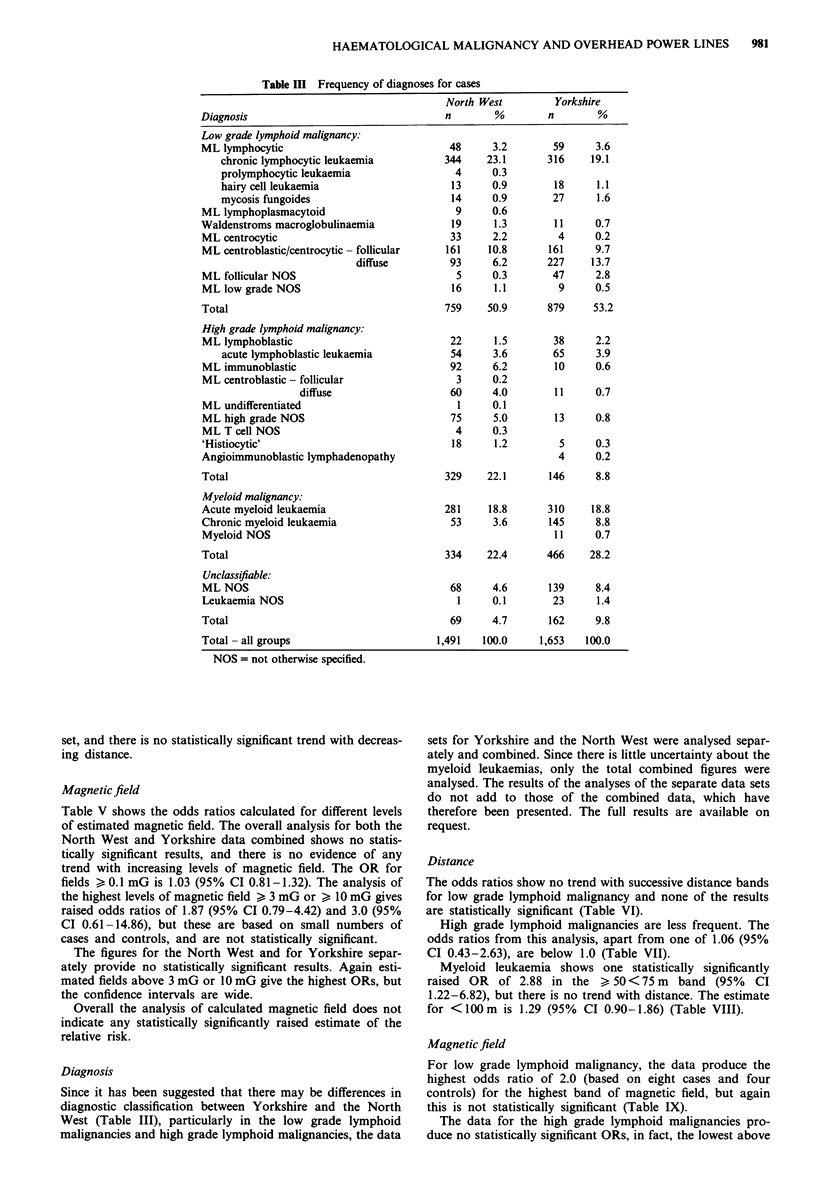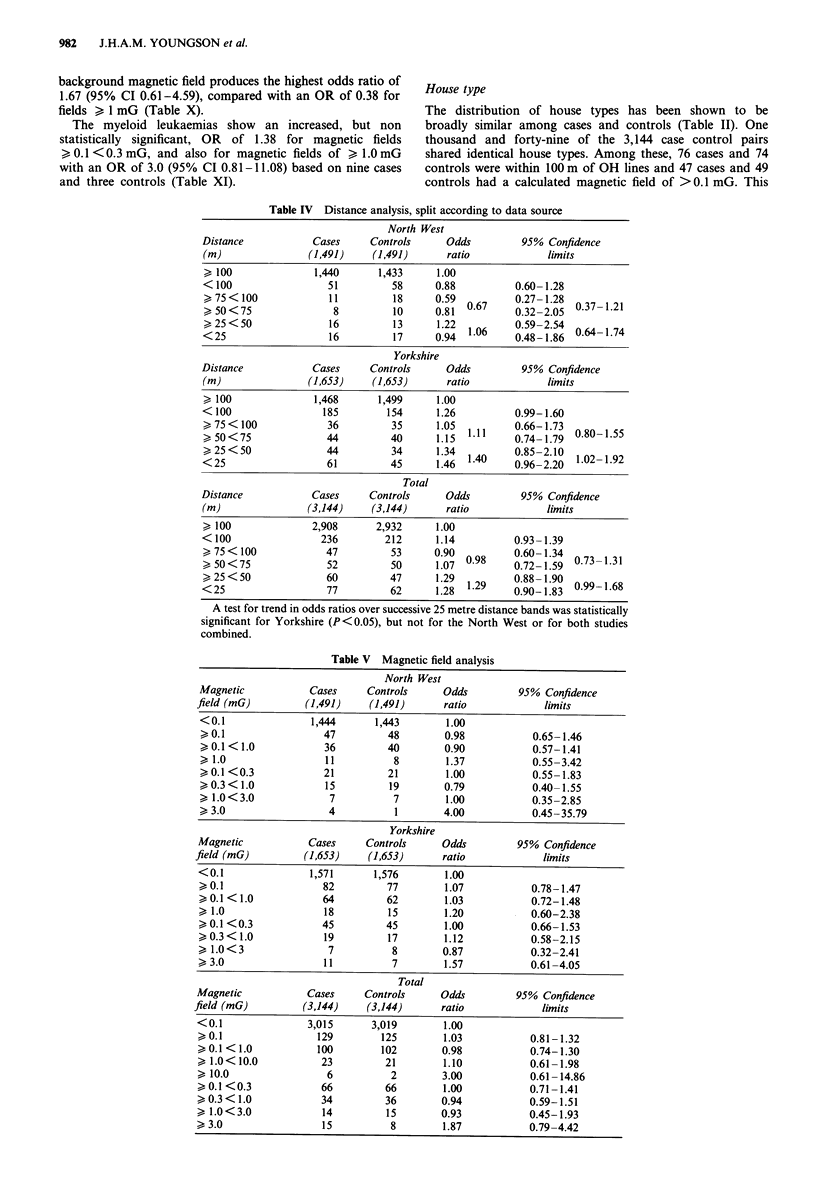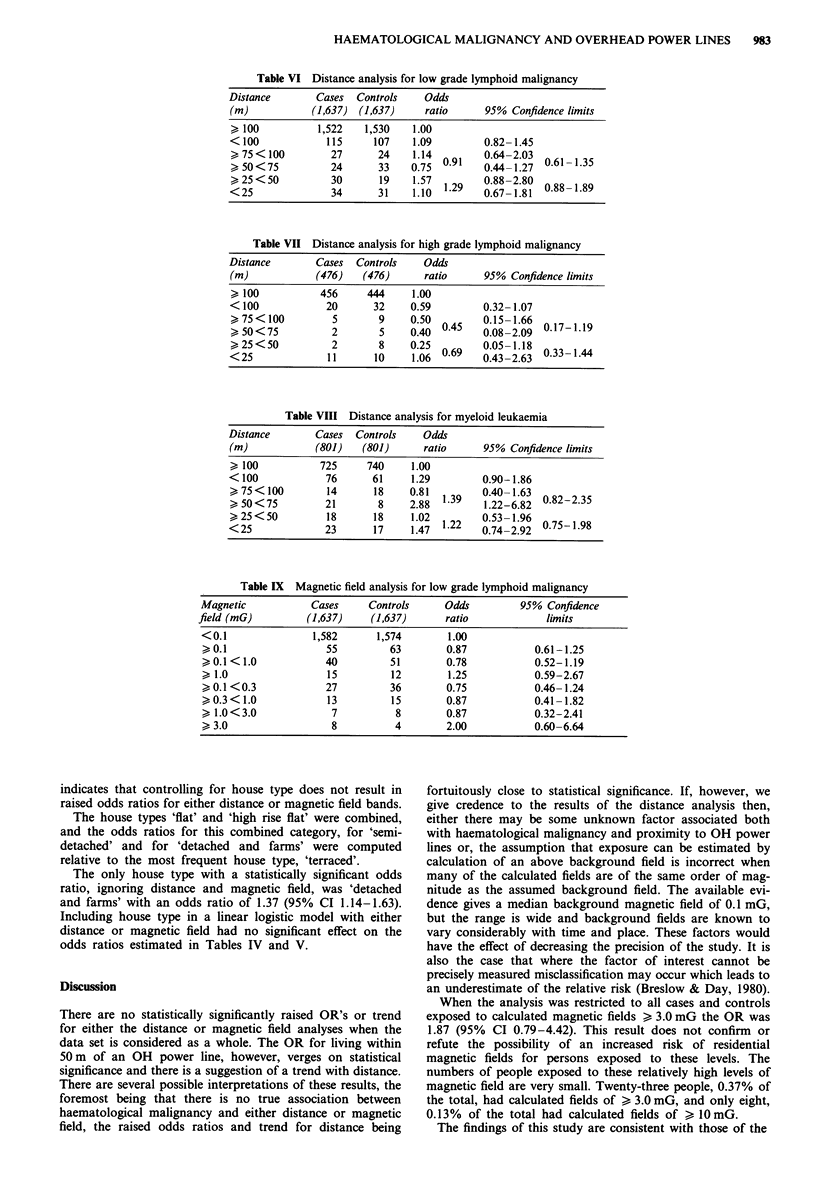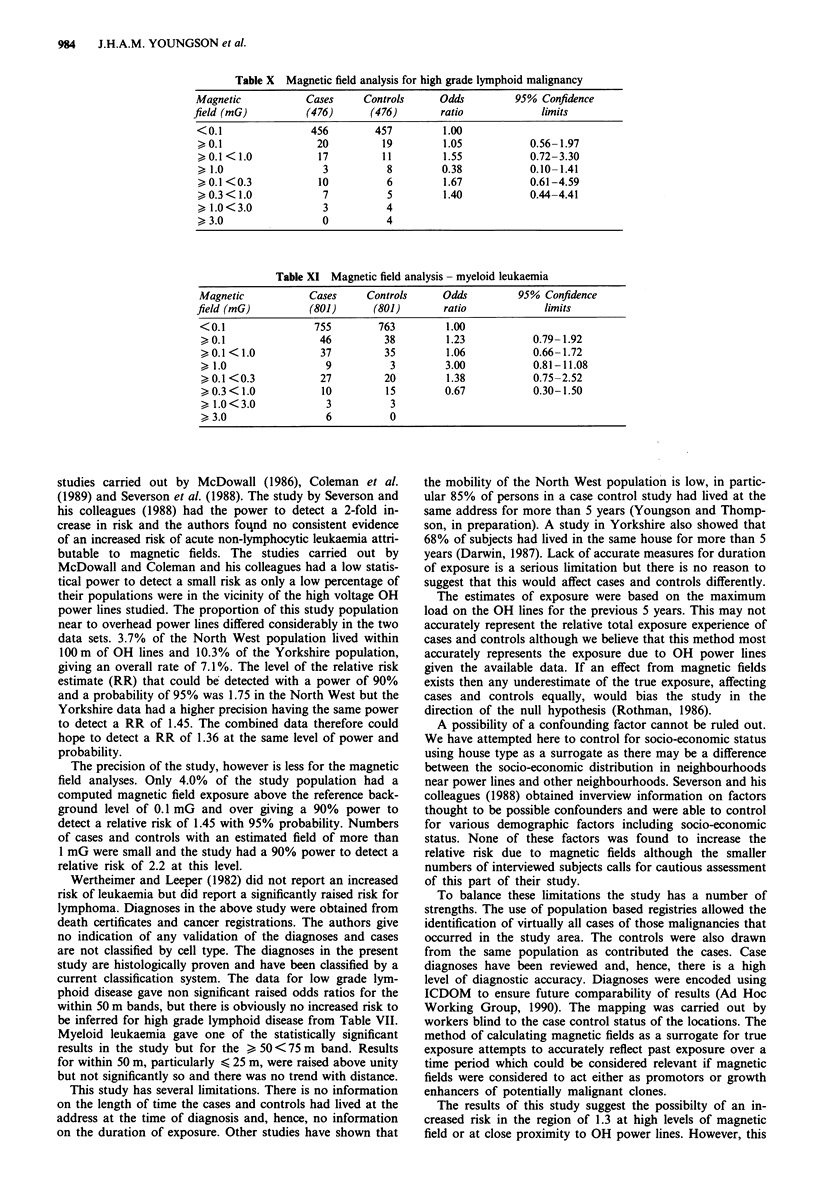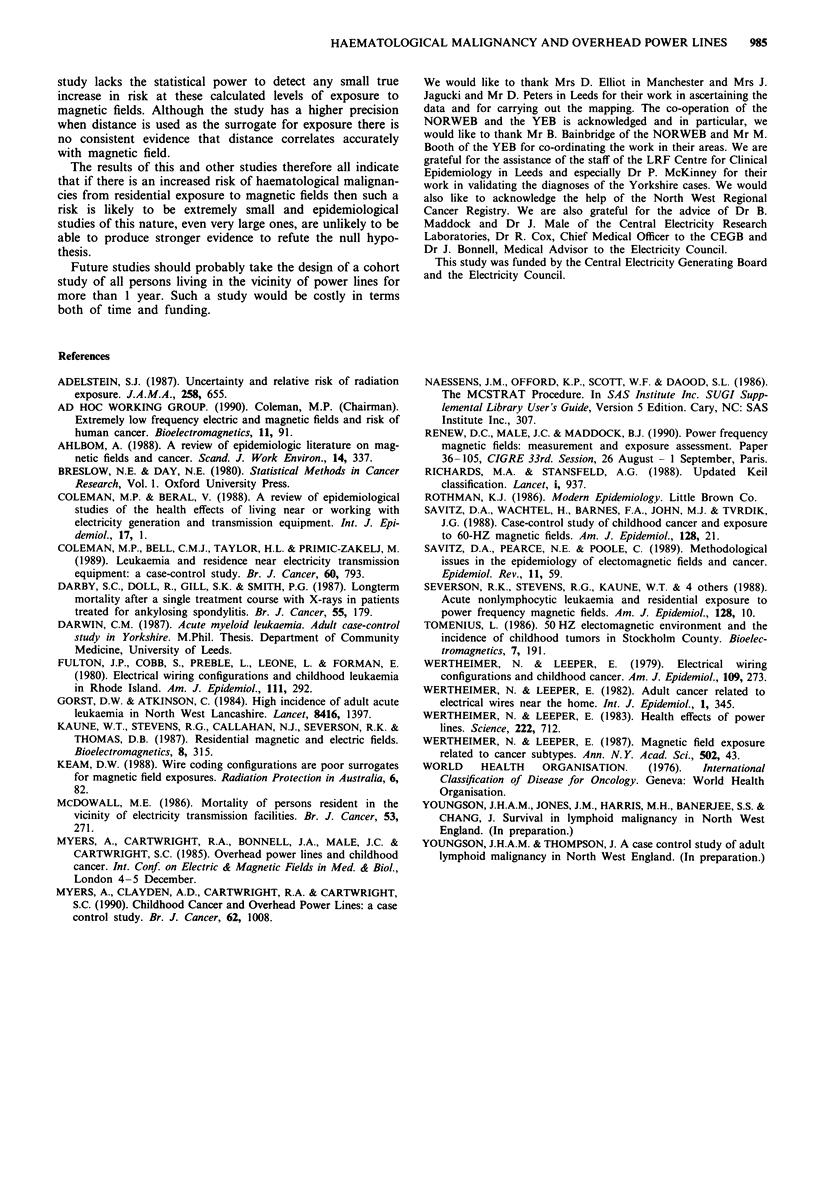# A case/control study of adult haematological malignancies in relation to overhead powerlines.

**DOI:** 10.1038/bjc.1991.214

**Published:** 1991-06

**Authors:** J. H. Youngson, A. D. Clayden, A. Myers, R. A. Cartwright

**Affiliations:** Department of Epidemiology and Social Oncology, University of Manchester, UK.

## Abstract

A population based case control study of adult haematological malignancy and distance from, and magnetic fields associated with, overhead (OH) power lines has been carried out in the North West and Yorkshire regions of England. Three-thousand, one hundred and forty-four cases with histologically proven disease were entered into the study. One control per case, matched for age, sex, year of diagnosis and health district of residence, was selected from hospital discharges. Seven per cent of cases and controls lived near to OH power lines as defined by the study protocol. The measure of exposure used was the calculated magnetic field strength at each of these addresses due to maximum load currents carried by OH power lines in the 5 years preceding diagnosis. The odds ratio (OR) for living within 50 m of an OH line was 1.29 with a 95% Confidence Interval (CI) of 0.99-1.68 but a chi 2 test for trend with distance was not statistically significant. The analysis of calculated magnetic fields, did not produce any statistically odds ratios. The OR for magnetic fields greater than or equal to 0.1 mG was 1.03 (95% CI 0.81 1.32). Analysis of magnetic fields greater than or equal to 3.0 mG gave an OR of 1.87 (95% CI 0.79 4.42), but this result is based on small numbers. No evidence was found for confounding by the type of dwelling which was used as a partial surrogate for socio-economic status.


Br. J. Cancer (1991), 63, 977-985 © Macmillan Press Ltd., 1991

### A case/control study of adult haematological malignancies in relation to overhead powerlines

J.H.A.M. Youngson[1,*], A.D. Clayden[2,†], A. Myers[3] & R.A. Cartwright[4]

[1]*Department of Epidemiology and Social Oncology, University of Manchester, Kinnaird Road, Manchester M20 9QL;* [2]*Department of Public Health Medicine, University of Leeds, Leeds LS2 9LN;* [3]*Department of Physics, University of Leeds, Leeds LS2 9JT; and* [4]*Leukaemia Research Fund Centre for Clinical Epidemiology at the University of Leeds, 17 Springfield Mount, Leeds LS2 9NG, UK.*

**Summary** A population based case control study of adult haematological malignancy and distance from, and magnetic fields associated with, overhead (OH) power lines has been carried out in the North West and Yorkshire regions of England. Three-thousand, one hundred and forty-four cases with histologically proven disease were entered into the study. One control per case, matched for age, sex, year of diagnosis and health district of residence, was selected from hospital discharges. Seven per cent of cases and controls lived near to OH power lines as defined by the study protocol. The measure of exposure used was the calculated magnetic field strength at each of these addresses due to maximum load currents carried by OH power lines in the 5 years preceding diagnosis. The odds ratio (OR) for living within 50 m of an OH line was 1.29 with a 95% Confidence Interval (CI) of 0.99–1.68, but a $\chi^2$ test for trend with distance was not statistically significant. The analysis of calculated magnetic fields did not produce any statistically significant odds ratios. The OR for magnetic fields $\geqslant$ 0.1 mG was 1.03 (95% CI 0.81–1.32). Analysis of magnetic fields $\geqslant$ 3.0 mG gave an OR of 1.87 (95% CI 0.79–4.42), but this result is based on small numbers. No evidence was found for confounding by the type of dwelling which was used as a partial surrogate for socio-economic status.

It has been suggested that exposure to alternating magnetic fields at the power supply frequency of 50–60 hertz may play a role in carcinogenesis. Wertheimer and Leeper (1979) were the first to report an association between residential wiring configurations and childhood cancer. Further studies of the relationship between residential magnetic fields and childhood cancer have given rise to conflicting results (Fulton, 1980; Tomenius, 1986; Savitz, 1988). In addition, a recent study of childhood cancer failed to show any significant association with magnetic fields due to overhead (OH) power lines (Myers *et al.*, 1990). Wertheimer and Leeper (1982) also described an association between adult cancer and the high current wiring configurations near cases' residences and postulated that patterns in the data suggested that the association was causally linked. The main cancer sites contributing to the reported elevation of risk were the nervous system, uterus, breast and lymphomas. Leukaemia did not appear to be associated with an increased risk . This study has been criticised with regard to study design (Ahlbom, 1988; Coleman & Beral, 1988). A subsequent publication by Wertheimer and Leeper (1987) suggested an association between leukaemia and magnetic fields, limited to the very old. Other studies of adult cancer carried out so far have failed to show any association with exposure to residential magnetic fields (McDowall, 1986; Severson *et al.*, 1988; Coleman *et al.*, 1989). Severson *et al.* (1988) found that neither directly measured magnetic fields nor the surrogate values based on the wiring configurations were associated with adult acute non-lymphocytic leukaemia. Coleman *et al.* (1989) also found no clear association between leukaemia and electricity distribution equipment. The studies of McDowell (1986) and Coleman *et al.* (1989) had a low statistical power to detect the effects of proximity to OH power lines because very low proportions of the study populations lived near the major OH power lines investigated. Most studies conducted in this field have used a surrogate for exposure to magnetic fields and have not measured them directly. Distance from OH power lines has been regarded as a proxy for exposure (McDowall, 1986; Coleman *et al.*, 1989), but distance takes no account of the current flowing or the physical arrangement of the conductors, both of which crucially determine the magnetic field. It has been claimed that the coding of wiring configurations used by Wertheimer and Leeper predicts measured fields (Kaune *et al.*, 1987), but Keam (1988) suggests that wire configurations are poor surrogates for field exposure. As the low voltage power supply in the UK is mainly carried by underground cables, differing markedly from the USA where the majority of the supply is carried on poles above ground, this method is not appropriate. Severson and his colleagues used wire configurations, coded both by the Wertheimer and Leeper system and also by a system devised by Kaune, for all cases and controls, but took direct measurements at only half of the homes at the time of interview as many subjects had moved house before interview. The correlation coefficient for the relationship between wire configurations coded by the Wertheimer and Leeper system and 24 h magnetic field measurements in their study was 0.41 suggesting only a weak correlation and indicating the limitations of using wire configurations as a proxy for measurements. However, several authors have suggested that wire configurations may be a better surrogate for historical exposure to magnetic fields than direct measurement at a single recent point in time (Savitz 1988, 1989; Wertheimer & Leeper, 1983). The repetition by Savitz of the Wertheimer and Leeper study in the Denver area 5 years later seemed to confirm that the magnetic fields overall changed little during this period (Keam, 1988), but such use of spot measurements would be inappropriate if there are large variations in magnetic fields over time. Such measures, therefore, are unlikely to adequately reflect past exposure (Ahlbom, 1988).

The present study is a population based case control study in the North West and Yorkshire Regional Health Authorities (RHA) areas of England to test the hypothesis that proximity to OH power lines at home is associated with the development of adult haematological malignancy and that

Correspondence: J.H.A.M. Youngson.

Present addresses: *Mersey Cancer Registry, Mersey Regional Health Authority, Hamilton House, 24 Pall Mall, Liverpool L3 6AB; †Information Services Directorate, Headquarters Offices, Leeds Western Health Authority, Great George Street, Leeds LS1 3EX, UK.

Abbreviations used: kV, kilovolts; V, volts; m, meter; mG, milligauss; CI, confidence interval; OR, odds ratio; OH, over head; RR, relative risk; ICDOM, International Classification of Disease for Oncology - Morphology; WHO, World Health Organisation; RHA, Regional Health Authority; NORWEB, North Western Electricity Board; YEB, Yorkshire Electricity Board; ML, Malignant Lymphoma.

Received 15 October 1990; and in revised form 28 January 1991.

any association is due to the magnetic fields produced by such lines. The study was designed to take criticisms of previous studies into account, particularly with regard to the approach to the determination of magnetic field exposure. Leukaemias and lymphomas were selected as the conditions which should offer the best chance of demonstrating a possible causal association for several reasons. These groups can be expected to have a relatively short latent period. The incidence of adult leukaemia following exposure to ionising radiation increases after 2 years and that of lymphomas over 5 to 25 years (Adelstein, 1987; Darby *et al.*, 1987) whereas the latency of adult epithelial tumours may be over 10 and up to 40 years. Lymphomas were reported to have an association with wire configurations by Wertheimer and Leeper (1982), and occupational studies have suggested an increased risk of acute non-lymphocytic leukaemia (Coleman & Beral, 1988). Leukaemia is a rare disease with relatively few cases available for study. Morphologically the cell types of certain subsets of non-Hodgkin's lymphomas are indistinguishable from the lymphoid leukaemias and the whole spectrum of the lymphoid malignancies is included in the current Kiel classification (Richards & Stansfeld, 1988). The inclusion of all the lymphoid malignancies increases the number of cases which can be accurately ascertained in a short period. A short recent study period was desirable to ensure the availability and high level of accuracy of power supply records which were needed for the exposure assessment.

The study correlates the exposure to magnetic fields from OH power lines with the magnetic field calculated at the home address of people in the study living near these lines, as discussed below. The field is calculated from the maximum current estimated to have been flowing in the line during the exposure period of 5 years prior to diagnosis.

It was estimated that 10% of the controls would live in the vicinity of OH power lines as defined by the study protocol and that the study would have a 90% power to detect an estimated relative risk of 1.3 with a probability of 95%.

### Technical background

Estimation of the exposure to magnetic fields presents formidable problems. There may be exposure to a background magnetic field from, for example, underground distribution cables and domestic wiring. There may also be exposure to additional fields from local sources such as domestic appliances. The approach followed in this study was to quantify exposure to those alternating magnetic fields which were additional to the usual background magnetic field. Measurements of this field at homes in the study were not undertaken because of the objections raised to such spot measurements, as discussed above, and also because of ethical and logistic considerations. Instead it was decided to use calculated values of the magnetic field. For most sources, the field cannot be calculated with any certainty. The precise details of complicated wiring arrangements may not be known and records of the current load may not be available. For OH lines, however, neither of these factors applies and the field can be calculated from the current and the distance between the conductors and between the line and the home. In this study we investigate the possible relationship between exposure to the additional field from this source and carcinogenesis.

The lines involved in the study included the overhead transmission system operated by the Central Electricity Generating Board (CEGB) at 400 and 275 kV and the high voltage (132, 66, 33, 11 and 6.6 kV) and low voltage (415 and 240 V) distribution systems operated by the North West Electricity Board (NORWEB) and the Yorkshire Electricity Board (YEB). The 240 V lines were single phase and all the rest were part of the three phase network, though in a very few cases only two phases were present. There were also a very few underground high voltage (275 kV) cables which had three conductors laid parallel to one another with a substantial distance between them. The fields in this case could be calculated in the same way as for OH lines and this has been done in this study. One such cable was identified in the study area but the fields calculated for the locations nearby were $<0.1$ milligauss (mG).

Myers *et al.* (1985) made spot measurements of the background field strengths in 44 homes in Yorkshire, which were not part of the main study, and reported a range between 0.01 mG and 4 mG with a median level of 0.15 mG. Calculations under maximum load conditions and assuming balanced phase currents, showed that fields due to overhead lines could not exceed a level of 0.1 mG at distances greater than: (a) 100 m from lines at 66 kV and below, (b) 250 m from single circuit 132 kV lines and (c) 500 m from dual circuit 132 kV lines, 275 kV lines and 400 kV lines. The phase configuration for some dual circuit 132 kV lines was such as to reduce the distance to within 250 m and a few short sections of dual circuit 400 kV lines were constructed in such a way that it was necessary to extend the distance to 1 km. In the present study, field calculations have therefore been made only for homes within these distances of the respective line types. More recent measurements by Renew *et al.* (1990) over several days in a similar number of homes both in London and Yorkshire have found time averaged background fields covering a range between 0.06 and 1.2 mG, with a median level of 0.3 mG. These results are essentially in agreement with the findings of Myers *et al.* (1985).

### Method

### *North West region*

#### *Cases*

All cases aged 15 years and over and diagnosed between January 1st 1983 and December 31st 1985 with non-Hodgkin's lymphoma, acute lymphoblastic leukaemia, chronic lymphocytic leukaemia, acute myeloid leukaemia and chronic myeloid leukaemia and resident within the North West RHA boundary were eligible for the study. Cases were ascertained from specific leukaemia and lymphoma registries (Youngson *et al.*, in preparation, Gorst, 1984) which have very high levels of diagnostic accuracy. Cases of lymphoid malignancy were classified by the current Kiel classification (Richards & Stansfeld, 1988) and coded using the International Classification of Disease for Oncology - Morphology, WHO 1976 (ICDOM). One thousand, five hundred and eleven cases were registered by 31 December 1985, five of which were found to be duplicate registrations. The date of the diagnostic pathology report was taken as the date of diagnosis.

#### *Controls*

One control per case was randomly selected from computer listings of all inpatient hospital discharges. A wide range of diagnostic groups was used, avoiding any form of neoplasm. Cancer registry records were checked to ensure that no control was registered. The clinician in charge was asked for permission to use the control data and was asked to confirm that the control did not have current diagnosed malignant disease. Controls were matched to within 3 years of the case date of birth, except where this would reduce the control age to below 15 when controls over the age of 15 were selected. The control date of hospital admission was matched to within 1 year of the case date of diagnosis and controls were also matched to health district of residence as a partial surrogate for urban/rural residence. Health Districts in England are administrative areas with populations ranging from 107,000 to 363,000 in the North West RHA and the Yorkshire RHA.

Thirteen cases remained without a matched control and two cases were found to be visitors to the region, therefore, 1,491 matched pairs were entered into the study.

*Yorkshire region*

*Cases*

All cases aged 15 years and over and diagnosed in the Yorkshire RHA between January 1st 1979 and December 31st 1985 with the same conditions as those listed for the North West were eligible for the study. The date of diagnosis was taken as the date of the first histology or cytology report.

Cases were obtained from four different sources in an attempt to identify a list of cases which was as complete as possible. The Yorkshire Cancer Registry was checked for cases with evidence of histopathology. Consultants holding clinics at hospitals in Yorkshire were circulated to identify additional new patients who had not been registered. The Leukaemia Research Fund's Centre for Clinical Epidemiology contributed cases collected for their own adult leukaemia and lymphoma case control studies, including an interview based study of living patients, these cases themselves often having been obtained from histopathological and haematological laboratory sources. Finally, the histopathology panel for lymphomas also provided cases. The same case was often identified from two or more of these sources and copies of registrations were excluded. The resulting list of cases was regarded as essentially complete. Only cases within the Yorkshire RHA were eligible for the study. A few cases from Harrogate, Northallerton, Scarborough and York Health Districts were excluded because their houses were found to be within the North Eastern Electricity Board area rather than the YEB area, and this caused difficulties in obtaining line information. Of the 1,770 cases identified originally, 1,653 remained in the study after exclusions.

*Controls*

One control was selected for each case, matched for sex, and for age to within 3 years, and where possible being diagnosed within the same year. The interview based study referred to above included two controls diagnosed as having had a non malignant disease in the same hospital, and this study was the major source of controls for the present study. Our cases were matched with those controls who had been diagnosed in the same health district, where this was possible. Controls from this study who were not used as matched for their own cases, were available for use as controls for other identified cases. The other source of controls was listings of people discharged from hospital, with cases being matched with people discharged from hospitals within the same health district as the case, but with a non malignant diagnosis (generally non urgent surgical or accident/trauma patients). The date of the controls' hospital admissions were matched to any year within the case diagnosis period (1979–1985), but with the age at diagnosis being consistent with the protocol.

The locations used for the study, for both regions, were the addresses at which the cases were living at the time of diagnosis and the addresses from which the controls were admitted to hospital for the control illness episode. The key date was the date of diagnosis for the cases and date of admission for controls.

Since it has been suggested that socio-economic status may be a confounding factor, cases and controls from both regions were classified by the type of house in which they lived, as a partial surrogate for socio-economic status.

*Mapping*

All stages of the mapping procedure were carried out without knowledge of the case control status. Addresses were first verified and 10 figure grid references obtained from Ordnance Survey maps held in the county records departments. The type of house, as identified on the maps, was classified as terraced if it adjoined similar properties on either side, as semi-detached if it adjoined a similar property on one side only, and as detached if the building did not adjoin any other dwelling. Multiple occupancy units were classified according to the number of units in the building, either up to 25 units or more than 25 units.

The addresses were then plotted on maps, held at the district NORWEB and YEB offices, showing the relevant OH power lines. Measurements, on the maps, were taken from the mid-point of the building to the nearest point on the OH line using standard metric scale rulers. These measurements, together with details of the OH line and location were recorded if the building was within the distances specified for the various types of OH line. Measurements were estimated to be accurate to within 5 m up to a distance of 500 m, and to within 10 m over greater distances.

*Exposure assessment*

The aim of the field calculations was to estimate the magnetic field strengths at each address due to the maximum load currents carried by nearby OH lines in the 5 years preceding the key date. The assumption was made that this was proportional to the exposure each person received during the 5 year period.

The loads for 400 and 275 kV lines were those obtaining at the period of maximum demand on the whole CEGB system in the relevant 5 years. The Area Boards were able to supply the actual maximum loads carried by individual lines in the 5 year period. For all lines above 33 kV the current at maximum demand was obtained from meters which recorded loads sustained for more than either a 20 min or 30 min period, depending on the type of meter installed. For all dual circuit lines additional information regarding the actual phase of each conductor was provided by the Boards. Indirect methods of estimating maximum demand for 11 kV, 6.6 kV and low voltage lines were agreed with engineers from the NORWEB and the YEB and the actual estimates were made by engineers who were not otherwise involved in the study.

The magnetic field was calculated at the centre of each dwelling and at a height of 1 m above ground level. The statutory minimum ground clearance, plus the working reserve, was assumed for each type of line. If an address was within the specified distance from more than one line, the total field was taken as the square root of the sum of the squares of the separate contributions of each line.

Details of field calculations are described elsewhere (Myers *et al.*, 1990).

*Analysis*

Matched pair analyses were done by conditional logistic regression for case control studies with categorical exposure variables (Breslow & Day, 1980) using the MCSTRAT procedure of the SAS statistical package (Naessens *et al.*, 1986). The main index of exposure used in the analysis was the calculated maximum 5 year magnetic field, with less than 0.1 mG as the referent category. Analyses were also carried out with distance categorised to $<25$ m, $\geqslant 25 \leqslant 50$ m, $>50 \leqslant 75$ m, $>75 \leqslant 100$ m, and $>100$ m, the last being used as the referent category. In this analysis, the distance to the nearest OH line within 100 m of the house was taken. The presence of any other OH lines in the vicinity of the house was ignored.

Analyses were also carried out for each of three diagnostic subsets. The lymphoid malignancies were divided into high grade disease and low grade disease according to the Kiel classification and the myeloid leukaemias were included as a separate subset.

A conditional logistic regression analysis was carried out also for distance and for magnetic field separately, allowing for the possible effect of the non matching variable 'house type'. House type was included in the regression equation by identifying house type as either 'semi-detached' or 'detached' or 'farms' (coded as 1), or 'other house type' (coded as 0).

### Results

### Total

Three thousand, two hundred and eighty-one cases with confirmed diagnoses were accrued into the study, 1,511 from the North West and 1,770 from Yorkshire. The numbers of these cases and their controls subsequently removed from the study depended on the order in which validation procedures were undertaken in the two regions.

The reasons for excluding cases and controls are shown in Table I. One thousand, four hundred and ninety-one pairs were available from the North West and 1,653 pairs from Yorkshire, giving a total, for both regions, of 3,144 matched pairs for analysis.

The 3,144 cases were made up of 1,737 (55.2%) males and 1,407 (44.8%) females with an age range of 15–99 and mean and median ages of 63. The sex and age distributions were very similar for both data sets (Table II). The distribution of case diagnoses is shown in Table III. There are several marked differences in the distribution of the non-Hodgkin's lymphomas in the two data sets. This may represent a real difference in the geographical distribution of certain cell types or may reflect differences in the way cases are classified by the two lymphoma panels. The major differences lie in the smaller numbers classified as ML immunoblastic and ML centroblastic, and the larger numbers classified as ML diffuse centroblastic-centrocytic in the Yorkshire region.

The North West and Yorkshire both had similar proportions of each of the five major house types, most house types being terraced (38%) or semi-detached (37%). Additionally, cases and controls had similar distributions, with cases having slightly more detached houses and farms (12%) than controls (9%), and fewer flats (10%) than controls (12%).

Ninety-three per cent of the cases and controls had houses which lay outside the specified distances, and therefore had an above background field of <0.1 mG. The calculated fields for those 448 (7%) houses within the specified distances ranged from 0 mG to 69 mG, with a median value of 0.04 mG, and 25th and 75th percentile values of 0.004 and 0.25.

### Distance

Analysis of the distance of locations from OH power lines for the combined North West and Yorkshire data sets shows no statistically significant results (Table IV), but the odds ratio (OR) of 1.14 for locations less than 100 m from lines approaches statistical significance with 95% Confidence Intervals (CI) of 0.93–1.39. The analysis by 25 m bands suggest a trend of increasing ORs over the successive 25 m distances, however a $\chi^2$ test for trend with distance was not statistically significant. Reanalysis with 50 m bands shows an OR of 1.29 for <50 m (95% CI 0.99–1.68) which verges on statistical significance. The analysis of the North West and Yorkshire data separately shows that the ORs for the Yorkshire region are consistently higher than those for the North West, but this difference does not achieve any statistical significance. The OR for locations less than 100 m from lines in Yorkshire was 1.26 and approaches statistical significance (95% CI 0.99–1.60). The analysis of 50 m bands just reaches statistical significance with an OR or 1.40 for <50 m (95% CI 1.02–1.92). The $\chi^2$ test for trend is also significant ($P<0.05$). The North West analysis gives ORs <1.0 in all cases except the $\geqslant 25 < 50$ m band and there is no apparent trend over distance.

These analyses suggest a possible increased estimate of the relative risk for locations within 50 m of OH power lines, though this is statistically non significant for the whole data

**Table I** Reasons for excluding cases and controls

| | *North West Case/control pairs* | *Yorkshire Case/control pairs* | *Total Case/control pairs* |
|---|---|---|---|
| Original total | 1,511 | 1,770 | 3,281 |
| Unmatched | 8 | 2 | 10 |
| Invalid match | 5 | 66 | 71 |
| Address not found/ invalid | 2 | 40 | 42 |
| Line load not known | 0 | 5 | 5 |
| Duplicate | 5 | 4 | 9 |
| Remaining total | 1,491 | 1,653 | 3,144 |

**Table II** Comparison of cases and controls with respect to sex, age and house-type

| | *North West* | | *Yorkshire* | | *Total* | |
|---|---|---|---|---|---|---|
| | *Cases (1,491)* | *Controls (1,491)* | *Cases (1,653)* | *Controls (1,653)* | *Cases (3,144)* | *Controls (3,144)* |
| Male | 825 | 825 | 912 | 912 | 1737 | 1737 |
| Female | 666 | 666 | 741 | 741 | 1407 | 1407 |
| *Age group* | | | | | | |
| 15–19 | 19 | 20 | 33 | 29 | 52 | 49 |
| 20–29 | 50 | 51 | 55 | 59 | 105 | 110 |
| 30–39 | 75 | 71 | 97 | 97 | 172 | 168 |
| 40–49 | 110 | 117 | 138 | 140 | 248 | 257 |
| 50–59 | 237 | 241 | 276 | 279 | 513 | 520 |
| 60–69 | 378 | 380 | 435 | 439 | 813 | 819 |
| 70–79 | 431 | 429 | 450 | 446 | 881 | 875 |
| 80–89 | 179 | 167 | 161 | 154 | 340 | 321 |
| 90+ | 12 | 15 | 8 | 10 | 20 | 25 |
| *House-type* | | | | | | |
| Terraced | 575 | 553 | 598 | 657 | 1173 | 1210 |
| Semi-detached | 538 | 544 | 644 | 617 | 1182 | 1161 |
| Detached and farms | 171 | 133 | 205 | 158 | 376 | 291 |
| Flat | 138 | 186 | 171 | 180 | 309 | 366 |
| High-rise flat | 42 | 36 | 32 | 36 | 74 | 72 |
| Caravans and other | 0 | 5 | 0 | 0 | 0 | 5 |
| Not known | 27 | 34 | 3 | 5 | 30 | 39 |

**Table III** Frequency of diagnoses for cases

| Diagnosis | North West n | North West % | Yorkshire n | Yorkshire % |
|---|---|---|---|---|
| *Low grade lymphoid malignancy:* | | | | |
| ML lymphocytic | 48 | 3.2 | 59 | 3.6 |
| chronic lymphocytic leukaemia | 344 | 23.1 | 316 | 19.1 |
| prolymphocytic leukaemia | 4 | 0.3 | | |
| hairy cell leukaemia | 13 | 0.9 | 18 | 1.1 |
| mycosis fungoides | 14 | 0.9 | 27 | 1.6 |
| ML lymphoplasmacytoid | 9 | 0.6 | | |
| Waldenstroms macroglobulinaemia | 19 | 1.3 | 11 | 0.7 |
| ML centrocytic | 33 | 2.2 | 4 | 0.2 |
| ML centroblastic/centrocytic – follicular | 161 | 10.8 | 161 | 9.7 |
| diffuse | 93 | 6.2 | 227 | 13.7 |
| ML follicular NOS | 5 | 0.3 | 47 | 2.8 |
| ML low grade NOS | 16 | 1.1 | 9 | 0.5 |
| Total | 759 | 50.9 | 879 | 53.2 |
| *High grade lymphoid malignancy:* | | | | |
| ML lymphoblastic | 22 | 1.5 | 38 | 2.2 |
| acute lymphoblastic leukaemia | 54 | 3.6 | 65 | 3.9 |
| ML immunoblastic | 92 | 6.2 | 10 | 0.6 |
| ML centroblastic – follicular | 3 | 0.2 | | |
| diffuse | 60 | 4.0 | 11 | 0.7 |
| ML undifferentiated | 1 | 0.1 | | |
| ML high grade NOS | 75 | 5.0 | 13 | 0.8 |
| ML T cell NOS | 4 | 0.3 | | |
| 'Histiocytic' | 18 | 1.2 | 5 | 0.3 |
| Angioimmunoblastic lymphadenopathy | | | 4 | 0.2 |
| Total | 329 | 22.1 | 146 | 8.8 |
| *Myeloid malignancy:* | | | | |
| Acute myeloid leukaemia | 281 | 18.8 | 310 | 18.8 |
| Chronic myeloid leukaemia | 53 | 3.6 | 145 | 8.8 |
| Myeloid NOS | | | 11 | 0.7 |
| Total | 334 | 22.4 | 466 | 28.2 |
| *Unclassifiable:* | | | | |
| ML NOS | 68 | 4.6 | 139 | 8.4 |
| Leukaemia NOS | 1 | 0.1 | 23 | 1.4 |
| Total | 69 | 4.7 | 162 | 9.8 |
| Total – all groups | 1,491 | 100.0 | 1,653 | 100.0 |

NOS = not otherwise specified.

set, and there is no statistically significant trend with decreasing distance.

### *Magnetic field*

Table V shows the odds ratios calculated for different levels of estimated magnetic field. The overall analysis for both the North West and Yorkshire data combined shows no statistically significant results, and there is no evidence of any trend with increasing levels of magnetic field. The OR for fields $\geqslant 0.1$ mG is 1.03 (95% CI 0.81–1.32). The analysis of the highest levels of magnetic field $\geqslant 3$ mG or $\geqslant 10$ mG gives raised odds ratios of 1.87 (95% CI 0.79–4.42) and 3.0 (95% CI 0.61–14.86), but these are based on small numbers of cases and controls, and are not statistically significant.

The figures for the North West and for Yorkshire separately provide no statistically significant results. Again estimated fields above 3 mG or 10 mG give the highest ORs, but the confidence intervals are wide.

Overall the analysis of calculated magnetic field does not indicate any statistically significantly raised estimate of the relative risk.

### *Diagnosis*

Since it has been suggested that there may be differences in diagnostic classification between Yorkshire and the North West (Table III), particularly in the low grade lymphoid malignancies and high grade lymphoid malignancies, the data sets for Yorkshire and the North West were analysed separately and combined. Since there is little uncertainty about the myeloid leukaemias, only the total combined figures were analysed. The results of the analyses of the separate data sets do not add to those of the combined data, which have therefore been presented. The full results are available on request.

### *Distance*

The odds ratios show no trend with successive distance bands for low grade lymphoid malignancy and none of the results are statistically significant (Table VI).

High grade lymphoid malignancies are less frequent. The odds ratios from this analysis, apart from one of 1.06 (95% CI 0.43–2.63), are below 1.0 (Table VII).

Myeloid leukaemia shows one statistically significantly raised OR of 2.88 in the $\geqslant 50 < 75$ m band (95% CI 1.22–6.82), but there is no trend with distance. The estimate for $< 100$ m is 1.29 (95% CI 0.90–1.86) (Table VIII).

### *Magnetic field*

For low grade lymphoid malignancy, the data produce the highest odds ratio of 2.0 (based on eight cases and four controls) for the highest band of magnetic field, but again this is not statistically significant (Table IX).

The data for the high grade lymphoid malignancies produce no statistically significant ORs, in fact, the lowest above background magnetic field produces the highest odds ratio of 1.67 (95% CI 0.61–4.59), compared with an OR of 0.38 for fields ⩾ 1 mG (Table X).

The myeloid leukaemias show an increased, but non statistically significant, OR of 1.38 for magnetic fields ⩾ 0.1 < 0.3 mG, and also for magnetic fields of ⩾ 1.0 mG with an OR of 3.0 (95% CI 0.81–11.08) based on nine cases and three controls (Table XI).

*House type*

The distribution of house types has been shown to be broadly similar among cases and controls (Table II). One thousand and forty-nine of the 3,144 case control pairs shared identical house types. Among these, 76 cases and 74 controls were within 100 m of OH lines and 47 cases and 49 controls had a calculated magnetic field of > 0.1 mG. This

**Table IV** Distance analysis, split according to data source

*North West*

| *Distance (m)* | *Cases (1,491)* | *Controls (1,491)* | *Odds ratio* | | *95% Confidence limits* | |
|---|---|---|---|---|---|---|
| ⩾ 100 | 1,440 | 1,433 | 1.00 | | | |
| < 100 | 51 | 58 | 0.88 | | 0.60–1.28 | |
| ⩾ 75 < 100 | 11 | 18 | 0.59 | 0.67 | 0.27–1.28 | 0.37–1.21 |
| ⩾ 50 < 75 | 8 | 10 | 0.81 | | 0.32–2.05 | |
| ⩾ 25 < 50 | 16 | 13 | 1.22 | 1.06 | 0.59–2.54 | 0.64–1.74 |
| < 25 | 16 | 17 | 0.94 | | 0.48–1.86 | |

*Yorkshire*

| *Distance (m)* | *Cases (1,653)* | *Controls (1,653)* | *Odds ratio* | | *95% Confidence limits* | |
|---|---|---|---|---|---|---|
| ⩾ 100 | 1,468 | 1,499 | 1.00 | | | |
| < 100 | 185 | 154 | 1.26 | | 0.99–1.60 | |
| ⩾ 75 < 100 | 36 | 35 | 1.05 | 1.11 | 0.66–1.73 | 0.80–1.55 |
| ⩾ 50 < 75 | 44 | 40 | 1.15 | | 0.74–1.79 | |
| ⩾ 25 < 50 | 44 | 34 | 1.34 | 1.40 | 0.85–2.10 | 1.02–1.92 |
| < 25 | 61 | 45 | 1.46 | | 0.96–2.20 | |

*Total*

| *Distance (m)* | *Cases (3,144)* | *Controls (3,144)* | *Odds ratio* | | *95% Confidence limits* | |
|---|---|---|---|---|---|---|
| ⩾ 100 | 2,908 | 2,932 | 1.00 | | | |
| < 100 | 236 | 212 | 1.14 | | 0.93–1.39 | |
| ⩾ 75 < 100 | 47 | 53 | 0.90 | 0.98 | 0.60–1.34 | 0.73–1.31 |
| ⩾ 50 < 75 | 52 | 50 | 1.07 | | 0.72–1.59 | |
| ⩾ 25 < 50 | 60 | 47 | 1.29 | 1.29 | 0.88–1.90 | 0.99–1.68 |
| < 25 | 77 | 62 | 1.28 | | 0.90–1.83 | |

A test for trend in odds ratios over successive 25 metre distance bands was statistically significant for Yorkshire ($P < 0.05$), but not for the North West or for both studies combined.

**Table V** Magnetic field analysis

*North West*

| *Magnetic field (mG)* | *Cases (1,491)* | *Controls (1,491)* | *Odds ratio* | *95% Confidence limits* |
|---|---|---|---|---|
| < 0.1 | 1,444 | 1,443 | 1.00 | |
| ⩾ 0.1 | 47 | 48 | 0.98 | 0.65–1.46 |
| ⩾ 0.1 < 1.0 | 36 | 40 | 0.90 | 0.57–1.41 |
| ⩾ 1.0 | 11 | 8 | 1.37 | 0.55–3.42 |
| ⩾ 0.1 < 0.3 | 21 | 21 | 1.00 | 0.55–1.83 |
| ⩾ 0.3 < 1.0 | 15 | 19 | 0.79 | 0.40–1.55 |
| ⩾ 1.0 < 3.0 | 7 | 7 | 1.00 | 0.35–2.85 |
| ⩾ 3.0 | 4 | 1 | 4.00 | 0.45–35.79 |

*Yorkshire*

| *Magnetic field (mG)* | *Cases (1,653)* | *Controls (1,653)* | *Odds ratio* | *95% Confidence limits* |
|---|---|---|---|---|
| < 0.1 | 1,571 | 1,576 | 1.00 | |
| ⩾ 0.1 | 82 | 77 | 1.07 | 0.78–1.47 |
| ⩾ 0.1 < 1.0 | 64 | 62 | 1.03 | 0.72–1.48 |
| ⩾ 1.0 | 18 | 15 | 1.20 | 0.60–2.38 |
| ⩾ 0.1 < 0.3 | 45 | 45 | 1.00 | 0.66–1.53 |
| ⩾ 0.3 < 1.0 | 19 | 17 | 1.12 | 0.58–2.15 |
| ⩾ 1.0 < 3 | 7 | 8 | 0.87 | 0.32–2.41 |
| ⩾ 3.0 | 11 | 7 | 1.57 | 0.61–4.05 |

*Total*

| *Magnetic field (mG)* | *Cases (3,144)* | *Controls (3,144)* | *Odds ratio* | *95% Confidence limits* |
|---|---|---|---|---|
| < 0.1 | 3,015 | 3,019 | 1.00 | |
| ⩾ 0.1 | 129 | 125 | 1.03 | 0.81–1.32 |
| ⩾ 0.1 < 1.0 | 100 | 102 | 0.98 | 0.74–1.30 |
| ⩾ 1.0 < 10.0 | 23 | 21 | 1.10 | 0.61–1.98 |
| ⩾ 10.0 | 6 | 2 | 3.00 | 0.61–14.86 |
| ⩾ 0.1 < 0.3 | 66 | 66 | 1.00 | 0.71–1.41 |
| ⩾ 0.3 < 1.0 | 34 | 36 | 0.94 | 0.59–1.51 |
| ⩾ 1.0 < 3.0 | 14 | 15 | 0.93 | 0.45–1.93 |
| ⩾ 3.0 | 15 | 8 | 1.87 | 0.79–4.42 |

**Table VI** Distance analysis for low grade lymphoid malignancy

| *Distance (m)* | *Cases (1,637)* | *Controls (1,637)* | *Odds ratio* | | *95% Confidence limits* | |
|---|---|---|---|---|---|---|
| ≥100 | 1,522 | 1,530 | 1.00 | | | |
| <100 | 115 | 107 | 1.09 | | 0.82–1.45 | |
| ≥75<100 | 27 | 24 | 1.14 | 0.91 | 0.64–2.03 | 0.61–1.35 |
| ≥50<75 | 24 | 33 | 0.75 | | 0.44–1.27 | |
| ≥25<50 | 30 | 19 | 1.57 | 1.29 | 0.88–2.80 | 0.88–1.89 |
| <25 | 34 | 31 | 1.10 | | 0.67–1.81 | |

**Table VII** Distance analysis for high grade lymphoid malignancy

| *Distance (m)* | *Cases (476)* | *Controls (476)* | *Odds ratio* | | *95% Confidence limits* | |
|---|---|---|---|---|---|---|
| ≥100 | 456 | 444 | 1.00 | | | |
| <100 | 20 | 32 | 0.59 | | 0.32–1.07 | |
| ≥75<100 | 5 | 9 | 0.50 | 0.45 | 0.15–1.66 | 0.17–1.19 |
| ≥50<75 | 2 | 5 | 0.40 | | 0.08–2.09 | |
| ≥25<50 | 2 | 8 | 0.25 | 0.69 | 0.05–1.18 | 0.33–1.44 |
| <25 | 11 | 10 | 1.06 | | 0.43–2.63 | |

**Table VIII** Distance analysis for myeloid leukaemia

| *Distance (m)* | *Cases (801)* | *Controls (801)* | *Odds ratio* | | *95% Confidence limits* | |
|---|---|---|---|---|---|---|
| ≥100 | 725 | 740 | 1.00 | | | |
| <100 | 76 | 61 | 1.29 | | 0.90–1.86 | |
| ≥75<100 | 14 | 18 | 0.81 | 1.39 | 0.40–1.63 | 0.82–2.35 |
| ≥50<75 | 21 | 8 | 2.88 | | 1.22–6.82 | |
| ≥25<50 | 18 | 18 | 1.02 | 1.22 | 0.53–1.96 | 0.75–1.98 |
| <25 | 23 | 17 | 1.47 | | 0.74–2.92 | |

**Table IX** Magnetic field analysis for low grade lymphoid malignancy

| *Magnetic field (mG)* | *Cases (1,637)* | *Controls (1,637)* | *Odds ratio* | *95% Confidence limits* |
|---|---|---|---|---|
| <0.1 | 1,582 | 1,574 | 1.00 | |
| ≥0.1 | 55 | 63 | 0.87 | 0.61–1.25 |
| ≥0.1<1.0 | 40 | 51 | 0.78 | 0.52–1.19 |
| ≥1.0 | 15 | 12 | 1.25 | 0.59–2.67 |
| ≥0.1<0.3 | 27 | 36 | 0.75 | 0.46–1.24 |
| ≥0.3<1.0 | 13 | 15 | 0.87 | 0.41–1.82 |
| ≥1.0<3.0 | 7 | 8 | 0.87 | 0.32–2.41 |
| ≥3.0 | 8 | 4 | 2.00 | 0.60–6.64 |

indicates that controlling for house type does not result in raised odds ratios for either distance or magnetic field bands.

The house types 'flat' and 'high rise flat' were combined, and the odds ratios for this combined category, for 'semi-detached' and for 'detached and farms' were computed relative to the most frequent house type, 'terraced'.

The only house type with a statistically significant odds ratio, ignoring distance and magnetic field, was 'detached and farms' with an odds ratio of 1.37 (95% CI 1.14–1.63). Including house type in a linear logistic model with either distance or magnetic field had no significant effect on the odds ratios estimated in Tables IV and V.

### Discussion

There are no statistically significantly raised OR's or trend for either the distance or magnetic field analyses when the data set is considered as a whole. The OR for living within 50 m of an OH power line, however, verges on statistical significance and there is a suggestion of a trend with distance. There are several possible interpretations of these results, the foremost being that there is no true association between haematological malignancy and either distance or magnetic field, the raised odds ratios and trend for distance being fortuitously close to statistical significance. If, however, we give credence to the results of the distance analysis then, either there may be some unknown factor associated both with haematological malignancy and proximity to OH power lines or, the assumption that exposure can be estimated by calculation of an above background field is incorrect when many of the calculated fields are of the same order of magnitude as the assumed background field. The available evidence gives a median background magnetic field of 0.1 mG, but the range is wide and background fields are known to vary considerably with time and place. These factors would have the effect of decreasing the precision of the study. It is also the case that where the factor of interest cannot be precisely measured misclassification may occur which leads to an underestimate of the relative risk (Breslow & Day, 1980).

When the analysis was restricted to all cases and controls exposed to calculated magnetic fields ≥3.0 mG the OR was 1.87 (95% CI 0.79–4.42). This result does not confirm or refute the possibility of an increased risk of residential magnetic fields for persons exposed to these levels. The numbers of people exposed to these relatively high levels of magnetic field are very small. Twenty-three people, 0.37% of the total, had calculated fields of ≥3.0 mG, and only eight, 0.13% of the total had calculated fields of ≥10 mG.

The findings of this study are consistent with those of the

**Table X** Magnetic field analysis for high grade lymphoid malignancy

| *Magnetic field (mG)* | *Cases (476)* | *Controls (476)* | *Odds ratio* | *95% Confidence limits* |
|---|---|---|---|---|
| <0.1 | 456 | 457 | 1.00 | |
| ⩾0.1 | 20 | 19 | 1.05 | 0.56–1.97 |
| ⩾0.1<1.0 | 17 | 11 | 1.55 | 0.72–3.30 |
| ⩾1.0 | 3 | 8 | 0.38 | 0.10–1.41 |
| ⩾0.1<0.3 | 10 | 6 | 1.67 | 0.61–4.59 |
| ⩾0.3<1.0 | 7 | 5 | 1.40 | 0.44–4.41 |
| ⩾1.0<3.0 | 3 | 4 | | |
| ⩾3.0 | 0 | 4 | | |

**Table XI** Magnetic field analysis – myeloid leukaemia

| *Magnetic field (mG)* | *Cases (801)* | *Controls (801)* | *Odds ratio* | *95% Confidence limits* |
|---|---|---|---|---|
| <0.1 | 755 | 763 | 1.00 | |
| ⩾0.1 | 46 | 38 | 1.23 | 0.79–1.92 |
| ⩾0.1<1.0 | 37 | 35 | 1.06 | 0.66–1.72 |
| ⩾1.0 | 9 | 3 | 3.00 | 0.81–11.08 |
| ⩾0.1<0.3 | 27 | 20 | 1.38 | 0.75–2.52 |
| ⩾0.3<1.0 | 10 | 15 | 0.67 | 0.30–1.50 |
| ⩾1.0<3.0 | 3 | 3 | | |
| ⩾3.0 | 6 | 0 | | |

studies carried out by McDowall (1986), Coleman *et al.* (1989) and Severson *et al.* (1988). The study by Severson and his colleagues (1988) had the power to detect a 2-fold increase in risk and the authors found no consistent evidence of an increased risk of acute non-lymphocytic leukaemia attributable to magnetic fields. The studies carried out by McDowall and Coleman and his colleagues had a low statistical power to detect a small risk as only a low percentage of their populations were in the vicinity of the high voltage OH power lines studied. The proportion of this study population near to overhead power lines differed considerably in the two data sets. 3.7% of the North West population lived within 100 m of OH lines and 10.3% of the Yorkshire population, giving an overall rate of 7.1%. The level of the relative risk estimate (RR) that could be detected with a power of 90% and a probability of 95% was 1.75 in the North West but the Yorkshire data had a higher precision having the same power to detect a RR of 1.45. The combined data therefore could hope to detect a RR of 1.36 at the same level of power and probability.

The precision of the study, however is less for the magnetic field analyses. Only 4.0% of the study population had a computed magnetic field exposure above the reference background level of 0.1 mG and over giving a 90% power to detect a relative risk of 1.45 with 95% probability. Numbers of cases and controls with an estimated field of more than 1 mG were small and the study had a 90% power to detect a relative risk of 2.2 at this level.

Wertheimer and Leeper (1982) did not report an increased risk of leukaemia but did report a significantly raised risk for lymphoma. Diagnoses in the above study were obtained from death certificates and cancer registrations. The authors give no indication of any validation of the diagnoses and cases are not classified by cell type. The diagnoses in the present study are histologically proven and have been classified by a current classification system. The data for low grade lymphoid disease gave non significant raised odds ratios for the within 50 m bands, but there is obviously no increased risk to be inferred for high grade lymphoid disease from Table VII. Myeloid leukaemia gave one of the statistically significant results in the study but for the ⩾50<75 m band. Results for within 50 m, particularly ⩽25 m, were raised above unity but not significantly so and there was no trend with distance.

This study has several limitations. There is no information on the length of time the cases and controls had lived at the address at the time of diagnosis and, hence, no information on the duration of exposure. Other studies have shown that the mobility of the North West population is low, in particular 85% of persons in a case control study had lived at the same address for more than 5 years (Youngson and Thompson, in preparation). A study in Yorkshire also showed that 68% of subjects had lived in the same house for more than 5 years (Darwin, 1987). Lack of accurate measures for duration of exposure is a serious limitation but there is no reason to suggest that this would affect cases and controls differently.

The estimates of exposure were based on the maximum load on the OH lines for the previous 5 years. This may not accurately represent the relative total exposure experience of cases and controls although we believe that this method most accurately represents the exposure due to OH power lines given the available data. If an effect from magnetic fields exists then any underestimate of the true exposure, affecting cases and controls equally, would bias the study in the direction of the null hypothesis (Rothman, 1986).

A possibility of a confounding factor cannot be ruled out. We have attempted here to control for socio-economic status using house type as a surrogate as there may be a difference between the socio-economic distribution in neighbourhoods near power lines and other neighbourhoods. Severson and his colleagues (1988) obtained inverview information on factors thought to be possible confounders and were able to control for various demographic factors including socio-economic status. None of these factors was found to increase the relative risk due to magnetic fields although the smaller numbers of interviewed subjects calls for cautious assessment of this part of their study.

To balance these limitations the study has a number of strengths. The use of population based registries allowed the identification of virtually all cases of those malignancies that occurred in the study area. The controls were also drawn from the same population as contributed the cases. Case diagnoses have been reviewed and, hence, there is a high level of diagnostic accuracy. Diagnoses were encoded using ICDOM to ensure future comparability of results (Ad Hoc Working Group, 1990). The mapping was carried out by workers blind to the case control status of the locations. The method of calculating magnetic fields as a surrogate for true exposure attempts to accurately reflect past exposure over a time period which could be considered relevant if magnetic fields were considered to act either as promotors or growth enhancers of potentially malignant clones.

The results of this study suggest the possibilty of an increased risk in the region of 1.3 at high levels of magnetic field or at close proximity to OH power lines. However, this study lacks the statistical power to detect any small true increase in risk at these calculated levels of exposure to magnetic fields. Although the study has a higher precision when distance is used as the surrogate for exposure there is no consistent evidence that distance correlates accurately with magnetic field.

The results of this and other studies therefore all indicate that if there is an increased risk of haematological malignancies from residential exposure to magnetic fields then such a risk is likely to be extremely small and epidemiological studies of this nature, even very large ones, are unlikely to be able to produce stronger evidence to refute the null hypothesis.

Future studies should probably take the design of a cohort study of all persons living in the vicinity of power lines for more than 1 year. Such a study would be costly in terms both of time and funding.

We would like to thank Mrs D. Elliot in Manchester and Mrs J. Jagucki and Mr D. Peters in Leeds for their work in ascertaining the data and for carrying out the mapping. The co-operation of the NORWEB and the YEB is acknowledged and in particular, we would like to thank Mr B. Bainbridge of the NORWEB and Mr M. Booth of the YEB for co-ordinating the work in their areas. We are grateful for the assistance of the staff of the LRF Centre for Clinical Epidemiology in Leeds and especially Dr P. McKinney for their work in validating the diagnoses of the Yorkshire cases. We would also like to acknowledge the help of the North West Regional Cancer Registry. We are also grateful for the advice of Dr B. Maddock and Dr J. Male of the Central Electricity Research Laboratories, Dr R. Cox, Chief Medical Officer to the CEGB and Dr J. Bonnell, Medical Advisor to the Electricity Council.

This study was funded by the Central Electricity Generating Board and the Electricity Council.

### References

ADELSTEIN, S.J. (1987). Uncertainty and relative risk of radiation exposure. *J.A.M.A.*, **258**, 655.

AD HOC WORKING GROUP. (1990). Coleman, M.P. (Chairman). Extremely low frequency electric and magnetic fields and risk of human cancer. *Bioelectromagnetics*, **11**, 91.

AHLBOM, A. (1988). A review of epidemiologic literature on magnetic fields and cancer. *Scand. J. Work Environ.*, **14**, 337.

BRESLOW, N.E. & DAY, N.E. (1980). *Statistical Methods in Cancer Research*, Vol. 1. Oxford University Press.

COLEMAN, M.P. & BERAL, V. (1988). A review of epidemiological studies of the health effects of living near or working with electricity generation and transmission equipment. *Int. J. Epidemiol.*, **17**, 1.

COLEMAN, M.P., BELL, C.M.J., TAYLOR, H.L. & PRIMIC-ZAKELJ, M. (1989). Leukaemia and residence near electricity transmission equipment: a case-control study. *Br. J. Cancer*, **60**, 793.

DARBY, S.C., DOLL, R., GILL, S.K. & SMITH, P.G. (1987). Longterm mortality after a single treatment course with X-rays in patients treated for ankylosing spondylitis. *Br. J. Cancer*, **55**, 179.

DARWIN, C.M. (1987). *Acute myeloid leukaemia. Adult case-control study in Yorkshire*. M.Phil. Thesis. Department of Community Medicine, University of Leeds.

FULTON, J.P., COBB, S., PREBLE, L., LEONE, L. & FORMAN, E. (1980). Electrical wiring configurations and childhood leukaemia in Rhode Island. *Am. J. Epidemiol.*, **111**, 292.

GORST, D.W. & ATKINSON, C. (1984). High incidence of adult acute leukaemia in North West Lancashire. *Lancet*, **8416**, 1397.

KAUNE, W.T., STEVENS, R.G., CALLAHAN, N.J., SEVERSON, R.K. & THOMAS, D.B. (1987). Residential magnetic and electric fields. *Bioelectromagnetics*, **8**, 315.

KEAM, D.W. (1988). Wire coding configurations are poor surrogates for magnetic field exposures. *Radiation Protection in Australia*, **6**, 82.

McDOWALL, M.E. (1986). Mortality of persons resident in the vicinity of electricity transmission facilities. *Br. J. Cancer*, **53**, 271.

MYERS, A., CARTWRIGHT, R.A., BONNELL, J.A., MALE, J.C. & CARTWRIGHT, S.C. (1985). Overhead power lines and childhood cancer. *Int. Conf. on Electric & Magnetic Fields in Med. & Biol.*, London 4–5 December.

MYERS, A., CLAYDEN, A.D., CARTWRIGHT, R.A. & CARTWRIGHT, S.C. (1990). Childhood Cancer and Overhead Power Lines: a case control study. *Br. J. Cancer*, **62**, 1008.

NAESSENS, J.M., OFFORD, K.P., SCOTT, W.F. & DAOOD, S.L. (1986). The MCSTRAT Procedure. In *SAS Institute Inc. SUGI Supplemental Library User's Guide*, Version 5 Edition. Cary, NC: SAS Institute Inc., 307.

RENEW, D.C., MALE, J.C. & MADDOCK, B.J. (1990). Power frequency magnetic fields: measurement and exposure assessment. Paper 36–105, *CIGRE 33rd. Session*, 26 August – 1 September, Paris.

RICHARDS, M.A. & STANSFELD, A.G. (1988). Updated Keil classification. *Lancet*, **i**, 937.

ROTHMAN, K.J. (1986). *Modern Epidemiology*. Little Brown Co.

SAVITZ, D.A., WACHTEL, H., BARNES, F.A., JOHN, M.J. & TVRDIK, J.G. (1988). Case-control study of childhood cancer and exposure to 60-HZ magnetic fields. *Am. J. Epidemiol.*, **128**, 21.

SAVITZ, D.A., PEARCE, N.E. & POOLE, C. (1989). Methodological issues in the epidemiology of electomagnetic fields and cancer. *Epidemiol. Rev.*, **11**, 59.

SEVERSON, R.K., STEVENS, R.G., KAUNE, W.T. & 4 others (1988). Acute nonlymphocytic leukaemia and residential exposure to power frequency magnetic fields. *Am. J. Epidemiol.*, **128**, 10.

TOMENIUS, L. (1986). 50 HZ electomagnetic environment and the incidence of childhood tumors in Stockholm County. *Bioelectromagnetics*, **7**, 191.

WERTHEIMER, N. & LEEPER, E. (1979). Electrical wiring configurations and childhood cancer. *Am. J. Epidemiol.*, **109**, 273.

WERTHEIMER, N. & LEEPER, E. (1982). Adult cancer related to electrical wires near the home. *Int. J. Epidemiol.*, **1**, 345.

WERTHEIMER, N. & LEEPER, E. (1983). Health effects of power lines. *Science*, **222**, 712.

WERTHEIMER, N. & LEEPER, E. (1987). Magnetic field exposure related to cancer subtypes. *Ann. N.Y. Acad. Sci.*, **502**, 43.

WORLD HEALTH ORGANISATION. (1976). *International Classification of Disease for Oncology*. Geneva: World Health Organisation.

YOUNGSON, J.H.A.M., JONES, J.M., HARRIS, M.H., BANERJEE, S.S. & CHANG, J. Survival in lymphoid malignancy in North West England. (In preparation.)

YOUNGSON, J.H.A.M. & THOMPSON, J. A case control study of adult lymphoid malignancy in North West England. (In preparation.)